# IgA Nephropathy: A Twenty Year Retrospective Single Center Experience

**DOI:** 10.4137/cmped.s2224

**Published:** 2009-02-18

**Authors:** Jacob Rube, Alexandra Peyser, Freya Tarapore, Bari Scheckner, Rachel Frank, Suzanne Vento, Cathy Hoffman, Douglas Charney, Elsa Valderamma, Beatrice Goilav, Howard Trachtman

**Affiliations:** 1 Department of Pediatrics. Division of Nephrology, Schneider Children’s Hospital, New Hyde Park, New York.; 2 Department of Pathology, North Shore-LIJ Health System, Long Island Campus of the Albert Einstein College of Medicine.

**Keywords:** IgA nephropathy, severity, retrospective, kidney biopsy

## Abstract

IgA nephropathy (IgAN) is a common glomerular disease whose etiology is unknown. Previous studies have described the clinical and laboratory features but none have specifically compared patients during different time periods. This 20 year retrospective study was performed to assess trends in the severity of IgAN from 1989–2008. We reviewed 57 patient charts that contained a confirmed biopsy diagnosis of IgAN and recorded data at the time of diagnosis and the final follow-up appointment. Clinical data included physical examination, urine, and blood tests. Patients were separated into two cohorts, Cohort 1 1989–1998 and Cohort 2 1999–2008. An increase in severity was noted in Cohort 2 based on a significantly higher Up/c and lower serum albumin level. Other prognostic indicators including GFRe, hematocrit, and glomerular injury score also demonstrated a trend towards more severe disease over the past 20 years. The patients in both Cohorts received similar treatments and had comparable renal function at the last follow-up visit. Based on our findings, we suggest that although a kidney biopsy is required to diagnose IgAN, the procedure may not be necessary in patients clinically suspected of having the disease but who have normal kidney function and minimal urine abnormalities.

## Introduction

IgA nephropathy (IgAN) is the most common cause of glomerular hematuria throughout the world.^1^,^2^ It is an immune-complex-mediated glomerulonephritis defined by the presence of mesangial IgA deposits accompanied by varying degrees of hypercellularity and glomerular sclerosis.^2^,^3^ Patients with IgAN present with recurrent episodes of gross hematuria with or without persistent microscopic hematuria or they may have just persistent microscopic hematuria.^3^–^5^ There may be variable presence of proteinuria.^4^,^6^ The family history is usually negative for other affected individuals.^1^,^2^ The etiology of the disease is unknown and there are no proven therapies.^2^,^7^,^8^

IgAN is characterized by a highly variable course ranging from a benign condition to rapid progression to renal failure. Approximately 20%–25% of patients will develop end stage kidney disease.^2^, ^6^ Recent studies attempted to correlate clinical and laboratory data with the severity of the disease.^8^–^13^

When IgAN is suspected clinically, it usually does not pose an immediate threat to clinical well being and there is no proven therapy for the disease. Under these circumstances, the use of a kidney biopsy may have evolved into a procedure that is reserved for more severe cases. Therefore, we performed this single center, retrospective chart review to test the hypotheses that: (1) there has been an increase in the severity of clinical presentation in patients with IgAN over the last 20 years, and (2) alterations in the treatment have resulted in improved outcomes.

## Patients and Methods

### Patients

This retrospective chart review was conducted in all pediatric patients who presented at Schneider Children’s Hospital with IgAN from 1989–2008. The general criterion for performing a kidney biopsy in these children was recurrent gross hematuria of glomerular origin and it was unchanged over the course of the study period. A database maintained at the Division of Pediatric Nephrology, which includes patient gender, age, and chief complaint, was scanned to identify all children with IgAN and their initial complaint. The database is set up to record only a single chief complaint at the first evaluation. The term entered was the complaint reported by the patient or the medical problem identified by the attending physician. The medical records were retrieved from the file of patients who were still being seen in the clinic or from off-site storage. A second database of biopsy procedures maintained by the Department of Pathology of North Shore-LIJ Health System containing the date of biopsy, patient date of birth, and the clinical diagnosis, was searched to identify all children with a diagnosis of IgAN. It was used to confirm the accuracy of the divisional database and ensure identification of all cases.

When available, the following data were extracted from each chart: age, gender, patient history, clinical symptoms, presence of gross hematuria, duration of the complaint, medications, family history, height (cm), weight (kg), blood pressure, pertinent findings on physical examination, initial dipstick urinalysis and microscopy, and blood tests including GFRe. The data from time of the diagnostic kidney biopsy and the most recent follow up were recorded.

### Pathology data

A diagnosis of IgAN was based on the finding of mesangial IgA deposits in the renal tissue sample that was obtained at the biopsy. Renal biopsies were assessed for severity of histopathological changes and graded on a three point scale: 1+, no interstitial fibrosis and no glomerular sclerosis; 2+, scattered foci of interstitial fibrosis and up to 25% of glomeruli with sclerosis; 3+, more wide spread foci of interstitial fibrosis and more than 25% of glomeruli showing sclerotic changes. The study focused on these features of the renal histopathology because they have been demonstrated to have the highest correlation with disease severity and long-term prognosis.^14^

### Statistics

Data analysis was based on calculations of the mean ± SD. A chi-square test was used to compare differences in proportions and a t-test was applied to compare means. Differences were considered statistically significant if the P value was less than 0.05.

All information was gathered on a preapproved form. Data were de-identified and coded by study number in accordance with the Health Insurance Portability and Accountability Act guidelines. This chart review was approved by the Institutional Review Board of North Shore-LIJ Health System.

## Results

Using the divisional database, a total of 65 records was identified that had a confirmed clinical diagnosis of IgAN. Another 17 records were identified from the Department of Pathology database as having IgAN. Of these 82, 71 (87%) charts were retrieved. 14 cases were excluded because the biopsy was done before 1989, no biopsy was recorded, or the clinical diagnosis of IgAN was not confirmed. Thus, this report is based on the resulting 57 cases.

The patients were split into two cohorts, those with a biopsy confirmation from 1989 to 1998 (Cohort 1, n = 29) and those between 1999 and June 2008 (Cohort 2, n = 28). There was one biopsy report in Cohort 2 that was unavailable because the procedure was done at another location. Information about the last follow up was missing in 2 patients in the first cohort and 9 in the second. The mean number of new cases per year during the study period was 3 (range: 1–7).

The clinical characteristics at the time of the diagnosis are summarized in the Table. The age, gender breakdown, presentation with microscopic hematuria and/or gross hematuria, and duration of symptoms were similar in the two periods. There was an increase in mean weight in period 2, consistent with the increased prevalence of overweight and obesity in the United States during the study period. There were statistically significant differences in the amount of proteinuria and in the serum albumin level, with slightly more abnormal values in patients evaluated in Cohort 2. There were no significant differences between the serum creatinine, GFRe, and hematocrit of patients in Cohort 1 and 2, although the trend was for mildly more abnormal values in the later period. Finally, the mean biopsy severity grading was 1.26 ± 0.49 for Cohort I and 1.52 ± 0.73 for Cohort II (P = 0.12). Eight patients had a biopsy severity score of 1.5 or greater in Cohort 1 versus 12 in Cohort 2 (P = 0.28). The finding of more than 1–3 glomerular crescents in the renal biopsy specimen was rare in both Cohorts and the mean percentage of affected glomeruli was 2.5 ± 4.7 in Cohort I versus 5.5 ± 10.6 in Cohort II (P = 0.22).

The clinical status at the most recent follow-up visit is also summarized in the Table. As expected, the mean duration of follow up in Cohort 1 was longer, 6 ± 3 versus 4 ± 2 years (P = 0.008). In Cohort 2, one patient developed end stage renal disease.

In Cohort 1, there were no patients who were prescribed prednisone, while 11 were given Vitamin E. In Cohort 2, 3 patients received prednisone and 7 were started on Vitamin E. Angiotensin converting enzyme inhibitors were prescribed to patients in both Cohorts with a Up/c > 1.

A high SBP (>95th percentile for age and gender), a low GFR (<90 mL/min/1.73 m^2^), and a high Up/c (> 1 mg/mg) were considered to be indicators of impaired renal function. At the last follow up, in Cohort 1, 13/26 (50%) had high SBP, 3/23 (13%) had a GFRe < 90, and 0/23 had an Up/c > 1. In the patients in Cohort 2, 6/19 (32%) had high SBP, 2/15 (13%) had a GFRe < 90, and 2/18 (11%) had an Up/c > 1. The differences in these outcomes between the two Cohorts were not significant.

## Discussion

The results of this retrospective single center study indicate that there has been a trend over the last 20 years towards modestly increased severity of the renal manifestations in children with IgAN. The chief clinical markers supportive of this assertion were higher levels of proteinuria and lower GFRe. There was a slightly higher score of biopsy severity in the Cohort 2 but the difference was marginal and did not substantially impact the management of individual patients.

The number of cases in this report is lower than in series reported from Pacific Rim countries where IgAN is especially prevalent and diagnosed based solely on urinary abnormalities. However, it is representative of other institutions in the United States and offers a contemporary picture of the disease. There was no increase in the annual number of new patients with IgAN over the 20 year period and the duration of symptoms before the biopsy was virtually identical. Interestingly, there was an increase in height and a statistically significant increase in body weight in the second cohort, consistent with secular trends in developed countries over the 20 year study period. Although obesity has been linked to renal disease, the most common histopathology findings are glomerulomegaly and lesions of focal segmental glomerulosclerosis.^15^ Thus, it is unlikely that either of these factors would have accounted for the slightly more severe renal manifestations of IgAN seen in Cohort 2. However, ongoing surveillance and further study is needed to ascertain whether increased prevalence of overweight and obesity is altering the severity of IgAN. Absent other explanations, we suggest that the changes reflect a tendency to defer a diagnostic kidney biopsy in children clinically suspected of having IgAN who have minimal proteinuria and a normal GFRe. In these cases, the decision to perform a kidney biopsy may be impacted by the need to provide parents with a firm diagnosis. Future studies will be needed to determine if the outcome in children with presumed IgAN differs from those with biopsy-confirmed disease.

At the last follow up visit, both patient groups exhibited similar clinical outcomes in terms of GFRe and the levels were not significantly different than the initial value. The reduction in GFRe at last follow-up in Cohort 1 was not significant (P = 0.10) The level of proteinuria was higher in the Cohort 2, but it was lower than the original value and was not significantly different from the level obtained in patients in Cohort 1 (P = 0.18). Only two patients in Cohort 2 had an Up/c > 1. The prevalence of the other two poor prognostic indices, namely high SBP and GFRe < 90 was the same in the two groups. Only one patient—in Cohort 2—developed renal failure. In the aggregate, our results suggest that IgAN is a relatively benign disease in children and adolescents. However, caution is advised in this regard because patients in Cohort 2 have been observed for a shorter period of time. Delineation of their clinical outcomes may require longer follow-up to more accurately define the prognosis.

Over the 20 year study period there was no consistent treatment protocol for patients with IgAN. Despite the modest increase in disease severity in patients in Cohort 2, minimal therapy was prescribed. Nonetheless, the general outcomes were similar and favorable in both groups. This reflects the generally benign manifestations of IgAN in both Cohorts and the lack of urgency to implement unproven therapies. The number of patients treated with prednisone and/or vitamin E and infrequently with more potent immunosuppressive medications remained the same in both Cohorts. This is consistent with the lack of published reports that have shown reproducible efficacy of any treatment except angiotensin converting enzyme inhibitors for pediatric patients with IgAN.^16^

Based on our findings, IgAN is a mild disease in pediatrics. Although a biopsy is important to confirm the etiology and establish the severity, it is reasonable to defer the procedure in patients clinically suspected of having IgAN who have normal kidney functions and minimal urine findings.

## Figures and Tables

**Table 1:** Clinical features.

	**Cohort 1 1989–1998 n = 29**	**Cohort 2 1999–2008 n = 28**
**First visit**		
Age (years)	11 ± 4	12 ± 4
M:F ratio	24:5	19:9
Duration of Symptoms (months)	11 ± 16	11 ± 11
Micro:Gross Hematuria	2:20	8:16
Height (cm)	142 ± 23	151 ± 21
Weight (kg)	40 ± 17	52 ± 22^*^
Up/c (mg/mg)	0.32 ± 0.32	1.13 ± 1.88^*^
GFRe (ml/min/1.73 m^2^)	132 ± 34	119 ± 40
Albumin (g/dL)	4.1 ± 0.3	3.8 ± 0.6^*^
Hct (vol%)	39 ± 5	38 ± 5
**Follow-up visit**		
Duration of follow up (years)	6 ± 3	4 ± 2
Number on Prednisone	0	3
Number on Vitamin E	11	7
SBP	117 ± 13	117 ± 20
DBP	67 ± 11	67 ± 17
Up/C	0.18 ± 0.18	0.97 ± 2.27
GFRe	118 ± 30	121 ± 34

Data are provided in mean ± SD.

* P < 0.05 compared to Cohort 1.

## Abbreviations

DBP: diastolic blood pressure;
GFRe: estimated glomerular filtration rate;
IgAN: Immunoglobulin A Nephropathy;
SBP: systolic blood pressure;
Up/c: Urine protein:creatinine ratio.
